# Suggestive evidence of *CYP4F2* gene polymorphisms with HAPE susceptibility in the Chinese Han population

**DOI:** 10.1371/journal.pone.0280136

**Published:** 2023-01-12

**Authors:** Lining Si, Haiyuan Wang, Rong Wang, Lhachen Tsering, Qifu Long, Yahui Jiang, Yun Yi, Yanli Zhao

**Affiliations:** 1 Department of Critical-Care Medicine, Affiliated Hospital of Qinghai University, Xining, Qinghai, China; 2 Department of Diabetes of Traditional Chinese Medicine, Qinghai Red Cross Hospital, Xining, Qinghai, China; 3 Medical College of Qinghai University, Xining, Qinghai, China; 4 Tibetan Medical College of Qinghai University, Xining, Qinghai, China; University of Colorado Denver Skaggs School of Pharmacy and Pharmaceutical Sciences, UNITED STATES

## Abstract

High altitude pulmonary edema (HAPE) is a common respiratory disease in the high altitude area, which is rapid and harmful. We firstly conducted a case-control study to assess the potential association of *CYP4F2* gene polymorphisms with HAPE susceptibility in the Chinese Han population. The study recruited 238 patients with HAPE and 230 healthy controls in Northwest China. Genomic DNA was extracted from blood samples, and gene polymorphisms were detected using the Agena MassARRAY platform. Odds ratios (ORs), 95% confidence intervals (95% CIs), and *P*-value were used to evaluate the relationship between HAPE risk and *CYP4F2* gene polymorphisms. Multi-factor dimension reduction (MDR) was used to assess the optimal interaction of *CYP4F2* gene polymorphisms on HAPE risk. We found rs3093193 was shown to reduce the risk of HAPE (OR = 0.70, 95% CI = 0.52–0.93, *P* = 0.014), while rs12459936 was increased the susceptibility to HAPE (OR = 2.08, 95% CI = 1.33–3.26, *P* = 0.001). Age stratified analysis revealed that rs3093193 and rs12459936 were correlated with HAPE risk in people at age > 32 years old, and rs3093193 and rs3093110 were correlated with the HAPE risk in people at age ≤ 32 years old. Gender stratification analysis was found that rs3093193, rs12459936, and rs3093110 were all related to HAPE risk in males. A combination of rs12459936 and rs3093110 was the best multi-loci model with the highest testing accuracy. Our study is the first to provide the association between *CYP4F2* gene polymorphisms and HAPE risk in the Chinese Han population.

## 1. Introduction

High altitude pulmonary edema (HAPE) is a hypoxia-induced non-cardiogenic pulmonary edema, which may have a further development when healthy individuals are first quickly ascended and exposed to altitude above 2500m [1]. HAPE, a kind of high permeability type of life-threatening pulmonary edema, is characterized by excessive pulmonary arterial hypertension [2,3] and its common symptoms are cough, dyspnea, chest tightness, and fatigue [4]. HAPE with its rapid occurrence and development can bloom into a coma or even life-threatening in an unexpected short time if left untreated [5]. However, the pathogenesis and influencing factors of HAPE remain to be studied. As is known to all, hypoxia induction is one of the principal elements in the occurrence of HAPE [6]. What’s more, hypoxia can also induce inflammation [7]. The inflammatory mediator is a vital and essential regulator of increasing vascular permeability and vasodilatation [8]. The increase of vascular permeability and vasoconstriction of diastolic imbalance is a crucial mechanism of acute high-altitude sickness. The bronchoalveolar fluid of patients with HAPE contains a large number of alveolar macrophages and detectable amounts of inflammatory factors such as leukotriene-B4 and other lipoxygenase products of arachidonic acid metabolism [9,10].

*CYP4F2*, which is a member of 4 subfamily F2 of the cytochrome P450 family, is a member of the cytochrome P450 enzyme superfamily as well. Gilroy *et al*. stated that the expression of *CYP4F2*, *CYP4F3*, and *CYP4A* family enzymes was upregulated during inflammation [11]. Additionally, *CYP4F2*-encoded enzymes start the process of inactivating and degrading leukotriene B4, a potent mediator of inflammation [12]. Furthermore, *CYP4F2* related pathway also includes arachidonic acid metabolism, which can be metabolize arachidonic acid to 20-hydroxyeicosatetraenoic acid (20-HETE). Arachidonic acid is also an important inflammatory cytokine, and its metabolic network is the core of the inflammatory metabolic network [13]. *CYP4F2*-derived 20-HETE is known to have the characteristics of prehypertension and angiogenesis promotions [14]. Therefore, we propose a reasonable hypothesis that the pathogenesis of HAPE is related to *CYP4F2*. Previously, polymorphisms of *CYP4F2* were reported to be linked with the susceptibility to pulmonary disease including lung cancer and chronic obstructive pulmonary disease [15,16], but not HAPE. Our study is the first to discuss the relationship between *CYP4F2* gene polymorphisms and HAPE risk in the Chinese Han population.

Here, we perform a hospital-based case-control study to explore the correlation of the four single nucleotide polymorphisms (SNPs) in *CYP4F2* at allele, genotype, and SNP-SNP interface with HAPE susceptibility among Chinese Han population.

## 2. Materials and methods

### 2.1. Participants

Patients with HAPE treated at the Affiliated Hospital of Qinghai University were enrolled as the case group. This research sought the consent of the Human Research Committee of Qinghai University (AF-RHEC-0018-01) and was conducted in accordance with the approved guidelines. During recruitment, the purpose of this study was well informed to the participants and written informed consent was obtained from each of them. The diagnosis of patients with HAPE was dependent on the standard criteria, including cough, dyspnea, cyanosis at rest, and imagological examination like X-ray radiograph, computed tomography (CT) of the patient chest or magnetic resonance imaging. All the patients with HAPE eventually showed that their chest has chest radiographic troves of infiltrates consistent with pulmonary edema. Healthy individuals who had a checkup in the hospital during the same period were recruited as the control group. Healthy controls were unrelated to each other and had no HAPE or related pulmonary diseases after exposure to 4000m altitude within 7 days. Each subject that we recruited in present study was healthy people without any previous history of cancers, cardiopulmonary and infectious diseases, or any other genetic diseases. Healthy controls were matched based on age. Finally, 238 patients with HAPE and 230 healthy individuals were enrolled in our study from 01/2019 to 12/2020. DNA extraction is performed immediately after the collected samples. Genotyping is performed after the collection and check of samples and clinical information (within 30 days after the completion of sample collection). All the subjects were the Han population in Northwest China and had no relation with each other.

### 2.2. SNP selection and genotyping

Peripheral blood samples of each subject (about 5 mL) were collected in tubes coated with ethylenediamine tetraacetic acid (EDTA). All samples were stored at -80°C. After being centrifugated, the whole blood cells were collected for further analysis [17]. Then, genomic DNA was extracted from the whole blood using a GoldMag whole blood genomic DNA purification kit (GoldMag Co. Ltd., Xi an, China) and following the manufacturer’s guidelines, and DNA quantity was assessed utilizing the NanoDrop 2000C spectrophotometer (Thermo Scientific, Waltham, MA, USA). Four SNPs (rs2881766, rs9383951, rs9340799, and rs3020449) in *CYP4F2* were involved in our study based on the 1,000 Genomes Project (http://www.1000genomes.org/) and the dbSNP (https: //www. ncbi.nlm.nih.gov/projects/SNP/) databases. The minor allele frequency (MAF) value > 0.05 was also applied to select candidate SNPs. The potential function of SNPs in *CYP4F2* was identified using HaploReg v4.1 in **Table 1** (https://pubs.broadinstitute.org/mammals/haploreg/haploreg.php). Agena MassARRAY Assay Design 3.0 Software (San Diego, California, USA) was used to design the primers for amplification and single-based extension. The corresponding primers of the selected SNPs in this study are listed in **Table 2**. SNP genotyping was carried out by two laboratory personnel in a double-blinded manner using the Agena MassARRAY system (Agena, San Diego, CA, U.S.A.). Agena Bioscience TYPER version 4.0 software was used for data analysis. In order to verify the accuracy of genotyping, approximately 10% of the samples were randomly selected for repeated genotyping and the reproducibility was 100%.

**Table 1:** Functional annotation of the selected variants provided by HaploReg 4.1.

SNP_ID	Ref/Alt	AFR	AMR	ASN	EUR	Promoter	Enhancer	Motifs changed	Selected eQTL hits
		freq	freq	freq	freq	histone marks	histone marks		
rs3093193	C/G	0.15	0.34	0.21	0.35	/	/	CTCF, Rad21	19 hits
rs12459936	C/T	0.01	0.15	0.55	0.03	/	BLD	ERalpha-a, TCF11/MafG, ZID	4 hits
rs3093144	C/T	0.21	0.17	0.13	0.17	/	LIV	6 altered motifs	26 hits
rs3093110	A/G	0.24	0.18	0.07	0.16	LIV, GI	LIV	CACD, PRDM1, VDR	9 hits

SNP: Single nucleotide polymorphism; Ref: Reference; Alt: Alternation; AFR: African; AMR: American; ASN: Asian; EUR: European; eQTL: Expression quantitative trait loci.

**Table 2:** Primers used for this study.

SNP_ID	2nd—PCRP	1st—PCRP	UEP DIR	UEP SEQ
rs3093193	ACGTTGGATGGCCACATACACATTGATGGG	ACGTTGGATGGTGATGAGACTAGTGATCCC	F	GTTTAGATAAACAGCCACA
rs12459936	ACGTTGGATGAGAGGTCGCAGTAAGCTGAG	ACGTTGGATGGGTAACCATCATTCTGCTTC	F	CAGCCTGGGTGACAGAG
rs3093144	ACGTTGGATGAGGAGTCTCTCGTCCTTCTG	ACGTTGGATGGGGAAGAATTGTGGCAAAGG	F	AGTTAAAAAAAAAATCCTAGATACTT
rs3093110	ACGTTGGATGTCCTGTTATGAGGGTACAGC	ACGTTGGATGGTCTCATTGATAAGAGGGAG	R	CCGTCTCCCACTTCCAC

PCR: Polymerase chain reaction, UEP: Unextended mini-sequencing primer.

### 2.3. Functional enrichment analysis

The *CYP4F2*-other interactions in our study were predicted with STRING (https://string-db.org/). After STRING database analysis, we obtained the gene interaction related to file package, and then through the analysis of the Cytoscape software to generate gene interaction result. Gene function analysis at the gene ontology (GO) level and Kyoto Encyclopaedia of Genes and Genomes (KEGG) pathway were performed using R package clusterProfiler. Furthermore, the NCBI Gene (https://www.ncbi.nlm.nih.gov/gene/) were used to search for HAPE susceptible genes by entering the key words of ‘High altitude pulmonary edema and susceptibility’. Here, 39 genes were found. We mixed these genes and *CYP4F2* related genes to perform PPI, GO and KEGG.

### 2.4. Statistical analysis

The differences of basic characteristics between the cases and controls were compared using Student’s *t*-test or *χ*^*2*^ test. Hardy-Weinberg equilibrium (HWE) was used for selected SNPs among controls, and the differences in genotypes distribution between cases and controls were examined using *χ*^*2*^ test. We evaluated the relationship of four SNPs with HAPE risk based on the five different genetic models that A and a are used to represent the major and minor alleles, respectively: the allele (a vs. A), codominant (homozygote model: aa vs. AA; heterozygote model: Aa vs. AA), recessive (aa vs. AA + Aa), dominant (Aa + aa vs. AA), log-additive models. Logistic regression analysis was used to estimate odds ratios (ORs) and its corresponding 95% confidence intervals (CIs), adjusted for age and gender using the PLINK software. Stratified analysis was also performed by gender and age to find out the relationship between each SNP and HAPE risk in different subgroups. Multi-factor dimension reduction (MDR) was used to assess the optimal interaction of *CYP4F2* gene polymorphisms on HAPE risk. False-positive report probability (FPRP) analysis was applied to assess the significant relationship of concerns. FPRP threshold was set at 0.2 for the significant relationship under investigation. A two-sided *P*-value < 0.05 was considered statistically significant. All the statistical analyses were performed using the software SPSS software package (version 20.0; SPSS Inc., Chicago, IL, USA) for Windows [18].

## 3. Results

### 3.1. The basic characteristics of study subjects

A total of 468 subjects including 238 patients with HAPE (220 males and 18 females) and 230 healthy individuals (213 males and 17 females) were enrolled in this study. The mean age and standard deviation of the cases and controls were 32.35 ± 10.78 years and 33.45 ± 9.05 years, respectively. As shown in **Table 3**, there was no significant difference between the case and control groups in regards to the distribution of gender and age (*P* = 0.915 and 0.236, respectively).

**Table 3:** Characteristics of patients with HAPE and control participants.

Variables	Case	Control	*P—*value
Total	238	230	
Age			0.236^a^
Age (mean ± SD, years)	32.35 ± 10.78	33.45 ± 9.05	
≤ 32 years	125 (53%)	115 (50%)	
> 32 years	113 (47%)	115 (50%)	
Gender			0.915^b^
Male	220 (92%)	213 (93%)	
Female	18 (8%)	17 (7%)	

HAPE: High altitude pulmonary edema.

*P*^***a***^-value was obtained from independent sample *t*-test.

*P*^b^-value was obtained from Pearson’s *χ*^2^ test.

### 3.2. Basic information and preliminary statistics of the selected SNPs

The basic information of the four SNPs was presented in **Table 4**. We genotyped the four SNPs (rs3093193, rs12459936, rs3093144, and rs3093110), and their genotyping success rates were > 99.0%. The genotype distribution of all SNPs in the control group were in accordance with HWE (*P* > 0.05). The allele frequency of rs3093193 was significantly different between healthy controls and HAPE patients, and rs3093193 was significantly correlated with a reduced risk of HAPE (OR = 0.70, 95% CI = 0.52–0.93, *P* = 0.014). However, the other three SNPs (rs12459936, rs3093144, and rs3093110) were not significantly associated with HAPE risk in the allele model (*P* = 0.099, 0.252, and 0.050, respectively).

**Table 4:** Basic information and allele frequency of the selected SNPs in *CYP4F2* gene.

SNP	Chr	Position	Role	Alleles	Frequency (MAF)		*P—HWE*	Call rate (%)	OR (95% CI)	*P*—value
					Cases	Controls				
rs3093193	19	15881104	intronic	C/G	0.235	0.306	0.999	100.0%	0.70 (0.52–0.93)	0.014*
rs12459936	19	15882231	intronic	C/T	0.512	0.458	0.145	100.0%	1.24 (0.96–1.60)	0.099
rs3093144	19	15891487	intronic	C/T	0.149	0.177	0.252	99.8%	0.82 (0.58–1.16)	0.252
rs3093110	19	15896974	intronic	A/G	0.090	0.130	0.239	100.0%	0.66 (0.44–1.00)	0.050

SNP: Single nucleotide polymorphism; HWE: Hardy-Weinberg equilibrium; OR: Odds ratio; 95% CI: 95% confidence interval.

*P-*value was obtained from *χ*^*2*^ test; *P-value < 0.05 indicates statistical significance.

### 3.3. Associations between genotype frequencies and HAPE susceptibility

Multiple inheritance models (dominant, recessive, additive, and codominant) were applied for analyzing the association between each SNP and HAPE risk (**Table 5**). Among four *CYP4F2* polymorphisms, rs3093193 was found to reduce HAPE risk in the dominant (C/G-G/G vs. C/C: OR = 0.66, 95% CI = 0.46–0.95, *P* = 0.027) and log-additive (OR = 0.70, 95% CI = 0.52–0.94, *P* = 0.017) models. As for rs12459936, an increased risk of HAPE was found in the codominant (C/T vs. C/C: OR = 2.08, 95% CI = 1.33–3.26, *P* = 0.001) and dominant (C/T-T/T vs. C/C: OR = 1.88, 95% CI = 1.23–2.88, *P* = 0.003) models. The other two SNPs (rs3093144 and rs3093110) were not related to HAPE susceptibility (*P* > 0.05).

**Table 5:** Logistic regression analysis of the association between the SNPs in *CYP4F2* gene and HAPE risk.

Model	Genotype	Control (n, %)	Case (n, %)	Without adjustment		With adjustment	
				OR (95% CI)	*P* ^*a*^ *—value*	OR (95% CI)	*P* ^*b*^ *—value*
rs3093193							
Codominant	C/C	110 (47.8%)	139 (58.4%)	1		1	
	C/G	99 (43.0%)	86 (36.1%)	0.69 (0.47–1.01)	0.055	0.70 (0.48–1.03)	0.067
	G/G	21 (9.1%)	13 (5.5%)	0.49 (0.23–1.02)	0.057	0.49 (0.24–1.03)	0.058
Dominant	C/C	110 (47.8%)	139 (58.4%)	1		1	
	C/G-G/G	120 (42.2%)	99 (41.6%)	0.65 (0.45–0.94)	0.022*	0.66 (0.46–0.95)	0.027*
Recessive	C/C-C/G	209 (90.9%)	225 (94.5%)	1		1	
	G/G	21 (9.1%)	13 (5.5%)	0.58 (0.28–1.18)	0.130	0.57 (0.28–1.17)	0.127
Log-additive	---	---	---	0.69 (0.52–0.93)	0.015*	0.70 (0.52–0.94)	0.017*
rs12459936							
Codominant	C/C	73 (31.7%)	47 (19.7%)	1		1	
	C/T	103 (44.8%)	138 (58%)	2.08 (1.33–3.25)	0.001*	2.08 (1.33–3.26)	0.001*
	T/T	54 (23.5%)	53 (22.3%)	1.52 (0.90–2.58)	0.117	1.50 (0.88–2.55)	0.133
Dominant	C/C	73 (31.7%)	47 (19.7%)	1		1	
	C/T-T/T	157 (68.3%)	191 (80.3%)	1.89 (1.24–2.88)	0.003*	1.88 (1.23–2.88)	0.003*
Recessive	C/C-C/T	176 (76.5%)	185 (77.7%)	1		1	
	T/T	54 (23.5%)	53 (22.3%)	0.93 (0.61–1.44)	0.756	0.92 (0.60–1.42)	0.699
Log-additive	---	---	---	1.25 (0.96–1.63)	0.094	1.24 (0.96–1.62)	0.106
rs3093144							
Codominant	C/C	158 (69%)	175 (73.5%)	1		1	
	C/T	61 (26.6%)	55 (23.1%)	0.81 (0.53–1.24)	0.341	0.82 (0.54–1.25)	0.352
	T/T	10 (4.4%)	8 (3.4%)	0.72 (0.28–1.88)	0.504	0.74 (0.28–1.92)	0.533
Dominant	C/C	158 (69.0%)	175 (73.5%)	1		1	
	C/T-T/T	71 (31.0%)	63 (26.5%)	0.80 (0.54–1.20)	0.279	0.81 (0.54–1.21)	0.295
Recessive	C/C-C/T	219 (95.6%)	230 (96.6%)	1		1	
	T/T	10 (4.4%)	8 (3.4%)	0.76 (0.30–1.97)	0.574	0.78 (0.30–2.01)	0.604
Log-additive	---	---	---	0.83 (0.59–1.16)	0.273	0.84 (0.60–1.17)	0.293
rs3093110							
Codominant	A/A	176 (76.5%)	198 (83.2%)	1		1	
	A/G	48 (20.9%)	37 (15.5%)	0.69 (0.43–1.10)	0.118	0.69 (0.43–1.12)	0.132
	G/G	6 (2.6%)	3 (1.3%)	0.44 (0.11–1.80)	0.257	0.44 (0.11–1.78)	0.250
Dominant	A/A	176 (76.5%)	198 (83.2%)	1		1	
	A/G-G/G	54 (23.5%)	40 (16.8%)	0.66 (0.42–1.04)	0.073	0.66 (0.42–1.05)	0.080
Recessive	A/A-A/G	224 (97.4%)	235 (98.7%)	1		1	
	G/G	6 (2.6%)	3 (1.3%)	0.48 (0.12–1.93)	0.299	0.47 (0.12–1.90)	0.289
Log-additive	---	---	---	0.68 (0.45–1.02)	0.060	0.68 (0.46–1.02)	0.065

HAPE: High altitude pulmonary edema; SNP: Single nucleotide polymorphism; OR: Odds ratio; 95% CI: 95% confidence interval.

*P*^a^-value was calculated by logistic regression analysis; *P*^b^-value was calculated by logistic regression analysis with adjustments for gender and age.

*P-value < 0.05 indicates statistical significance.

### 3.4. Stratification analysis of *CYP4F2* gene polymorphisms and HAPE risk

Stratified analysis regarding the impact of *CYP4F2* gene polymorphisms on HAPE according to age was displayed in **Table 6**. The results indicated that rs3093193 was correlated with a decreased HAPE risk at age ≤ 32 years in the allele (OR = 0.63, *P* = 0.028), codominant (G/G vs. C/C: OR = 0.28, *P* = 0.024) and recessive (G/G vs. C/C-C/G: OR = 0.30, *P* = 0.027) models. Rs12459936 was related to enhance the HAPE risk at age ≤ 32 years in the allele (OR = 1.52, *P* = 0.022), codominant (C/T vs. C/C: OR = 2.92, *P* = 0.001; T/T vs. C/C: OR = 2.30, *P* = 0.027), dominant (C/T-T/T vs. C/C: OR = 2.69, *P* = 0.002) and log-additive (OR = 1.52, *P* = 0.026) models. Besides, two loci (rs3093193 and rs3093110) of *CYP4F2* were observed to be associated with the decreased HAPE risk at age > 32 years. Rs3093193 was correlated with a decreased HAPE risk at age > 32 years in the codominant model (C/G vs. C/C: OR = 0.57, *P* = 0.046). Rs3093110 was associated with a decreased HAPE risk at age > 32 years under the allele (OR = 0.53, *P* = 0.034), codominant (A/G vs. A/A: OR = 0.42, *P* = 0.013), dominant (A/G-G/G vs. A/A: OR = 0.44, *P* = 0.016), and log-additive (OR = 0.52, *P* = 0.034) models.

**Table 6:** Stratified analysis of *CYP4F2* gene polymorphisms between age and risk of HAPE.

SNP	Model	Genotype	Age ≤ 32 years				Age >32 years			
			control	case	OR (95% CI)	*P—value*	control	case	OR (95% CI)	*P—value*
rs3093193	Allele	C	158 (68.7%)	194 (77.6%)	1		161 (70.0%)	170 (75.2%)	1	
		G	72 (31.3%)	56 (22.4%)	0.63 (0.42–0.95)	0.028*	69 (30.0%)	56 (24.8%)	0.77 (0.51–1.16)	0.211
	Codominant	C/C	57 (49.6%)	74 (59.2%)	1		53 (46.1%)	65 (57.5%)	1	
		C/G	44 (38.3%)	46 (36.8%)	0.87 (0.50–1.52)	0.633	55 (47.8%)	40 (35.4%)	0.57 (0.33–0.99)	0.046*
		G/G	14 (12.2%)	5 (4%)	0.28 (0.10–0.85)	0.024*	7 (6.1%)	8 (7.1%)	0.91 (0.31–2.68)	0.858
	Dominant	C/C	57 (49.6%)	74 (59.2%)	1		53 (46.1%)	65 (57.5%)	1	
		C/G-G/G	58 (50.4%)	51 (40.8%)	0.72 (0.43–1.22)	0.225	62 (53.9%)	48 (42.5%)	0.61 (0.36–1.03)	0.065
	Recessive	C/C-C/G	101 (87.8%)	120 (96.0%)	1		108 (93.9%)	105 (92.9%)	1	
		G/G	14 (12.2%)	5 (4.0%)	0.30 (0.10–0.87)	0.027*	7 (6.1%)	8 (7.1%)	1.16 (0.40–3.34)	0.781
	Log-additive	---	---	---	0.67 (0.44–1.01)	0.056	---	---	0.74 (0.48–1.14)	0.167
rs12459936	Allele	C	129 (56.1%)	114 (45.6%)	1		120 (52.2%)	118 (52.2%)	1	
		T	101 (43.9%)	136 (54.4%)	1.52 (1.06–2.18)	0.022*	110 (47.8%)	108 (47.8%)	1.00 (0.69–1.44)	0.993
	Codominant	C/C	42 (36.5%)	22 (17.6%)	1		31 (27%)	25 (22.1%)	1	
		C/T	45 (39.1%)	70 (56%)	2.92 (1.52–5.63)	0.001*	58 (50.4%)	68 (60.2%)	1.51 (0.79–2.85)	0.211
		T/T	28 (24.3%)	33 (26.4%)	2.30 (1.10–4.83)	0.027*	26 (22.6%)	20 (17.7%)	0.99 (0.45–2.18)	0.979
	Dominant	C/C	42 (36.5%)	22 (17.6%)	1		31 (27.0%)	25 (22.1%)	1	
		C/T-T/T	73 (63.5%)	103 (82.4%)	2.69 (1.45–4.96)	0.002*	84 (73.0%)	88 (77.9%)	1.34 (0.73–2.48)	0.345
	Recessive	C/C-C/T	87 (75.7%)	92 (73.6%)	1		89 (78.4%)	93 (82.3%)	1	
		T/T	28 (24.3%)	33 (26.4%)	1.15 (0.63–2.09)	0.641	26 (22.6%)	20 (17.7%)	0.75 (0.39–1.43)	0.379
	Log-additive	---	---	---	1.52 (1.05–2.19)	0.026*	---	---	1.02 (0.69–1.5)	0.939
rs3093144	Allele	C	185 (80.4%)	216 (86.4%)	1		192 (84.2%)	189 (83.6%)	1	
		T	45 (19.6%)	34 (13.6%)	0.65 (0.40–1.05)	0.078	36 (15.8%)	37 (16.4%)	1.04 (0.63–1.72)	0.866
	Codominant	C/C	76 (66.1%)	93 (74.4%)	1		82 (71.9%)	82 (72.6%)	1	
		C/T	33 (28.7%)	30 (24%)	0.83 (0.46–1.51)	0.537	28 (24.6%)	25 (22.1%)	0.87 (0.47–1.63)	0.666
		T/T	6 (5.2%)	2 (1.6%)	0.3 (0.06–1.56)	0.153	4 (3.5%)	6 (5.3%)	1.48 (0.40–5.46)	0.559
	Dominant	C/C	76 (66.1%)	93 (74.4%)	1		82 (71.9%)	82 (72.6%)	1	
		C/T-T/T	39 (33.9%)	32 (25.6%)	0.74 (0.42–1.32)	0.313	32 (28.1%)	31 (27.4%)	0.95 (0.53–1.7)	0.857
	Recessive	C/C-C/T	109 (94.8%)	123 (98.4%)	1		110 (96.5%)	107 (94.7%)	1	
		T/T	6 (5.2%)	2 (1.6%)	0.32 (0.06–1.63)	0.170	4 (3.5%)	6 (5.3%)	1.53 (0.42–5.59)	0.523
	Log-additive	---	---	---	0.72 (0.44–1.17)	0.181	---	---	1.02 (0.64–1.64)	0.927
rs3093110	Allele	A	204 (88.7%)	226 (90.4%)	1		196 (85.2%)	207 (91.6%)	1	
		G	26 (11.3%)	24 (9.6%)	0.83 (0.46–1.5)	0.541	34 (14.8%)	19 (8.4%)	0.53 (0.29–0.96)	0.034*
	Codominant	A/A	93 (80.9%)	102 (81.6%)	1		83 (72.2%)	96 (85%)	1	
		A/G	18 (15.7%)	22 (17.6%)	1.10 (0.54–2.23)	0.787	30 (26.1%)	15 (13.3%)	0.42 (0.21–0.83)	0.013*
		G/G	4 (3.5%)	1 (0.8%)	0.19 (0.02–1.76)	0.143	2 (1.7%)	2 (1.8%)	0.85 (0.12–6.21)	0.869
	Dominant	A/A	93 (80.9%)	102 (81.6%)	1		83 (72.2%)	96 (85.0%)	1	
		A/G-G/G	22 (19.1%)	23 (18.4%)	0.92 (0.47–1.79)	0.801	32 (27.8%)	17 (15.0%)	0.44 (0.23–0.86)	0.016*
	Recessive	A/A-A/G	111 (96.5%)	124 (99.2%)	1		113 (98.3%)	111 (98.2%)	1	
		G/G	4 (3.5%)	1 (0.8%)	0.19 (0.02–1.72)	0.139	2 (1.7%)	2 (1.8%)	1.00 (0.14–7.31)	0.999
	Log-additive	---	---	---	0.81 (0.46–1.43)	0.462	---	---	0.52 (0.29–0.95)	0.034*

HAPE: High altitude pulmonary edema; SNP: Single nucleotide polymorphism; OR: Odds ratio; 95% CI: 95% confidence interval.

*P*-value was calculated by logistic regression analysis with adjustments for gender and age; *P-value < 0.05 indicates statistical significance.

Furthermore, we conducted another stratified analysis of gender adjusted for age as shown in **Table 7**. In males, rs3093193 was correlated with a decreased risk of HAPE in the allele (OR = 0.67, *P* = 0.010), codominant (C/G vs. C/C: OR = 0.65, *P* = 0.036), dominant (C/G-G/G vs. C/C: OR = 0.62, *P* = 0.015) and log-additive (OR = 0.67, *P* = 0.011) models. Rs12459936 was related to an increased risk of HAPE in the codominant (C/T vs. C/C: OR = 2.12, *P* = 0.002) and dominant (C/T-T/T vs. C/C: OR = 1.94, *P* = 0.003) models. Rs3093110 was related to a reduced HAPE risk in the allele (OR = 0.61, *P* = 0.026) and log-additive (OR = 0.63, *P* = 0.034) models. No significant correlation between the genotype of rs3093144 and HAPE risk was observed.

**Table 7:** Association between *CYP4F2* gene polymorphisms and HAPE risk in males.

SNP	Model	Genotype	Male			
			Control	Case	OR (95% CI)	*P—value*
rs3093193	Allele	C	295 (69.2%)	339 (77.0%)	1	
		G	131 (30.8%)	101 (23.0%)	0.67 (0.50–0.91)	0.010*
	Codominant	C/C	101 (47.4%)	131 (59.5%)	1	
		C/G	93 (43.7%)	77 (35%)	0.65 (0.44–0.97)	0.036*
		G/G	19 (8.9%)	12 (5.5%)	0.48 (0.22–1.04)	0.062
	Dominant	C/C	101 (47.4%)	131 (59.5%)	1	
		C/G-G/G	112 (52.6%)	89 (40.5%)	0.62 (0.42–0.91)	0.015*
	Recessive	C/C-C/G	194 (91.1%)	208 (94.5%)	1	
		G/G	19 (8.9%)	12 (5.5%)	0.57 (0.27–1.22)	0.147
	Log-additive	---	---	---	0.67 (0.50–0.91)	0.011*
rs12459936	Allele	C	233 (54.7%)	214 (48.6%)	1	
		T	193 (45.3%)	226 (51.4%)	1.28 (0.98–1.67)	0.074
	Codominant	C/C	68 (31.9%)	43 (19.5%)	1	
		C/T	97 (45.5%)	128 (58.2%)	2.12 (1.33–3.37)	0.002*
		T/T	48 (22.5%)	49 (22.3%)	1.60 (0.92–2.78)	0.097
	Dominant	C/C	68 (31.9%)	43 (19.5%)	1	
		C/T-T/T	145 (68.1%)	177 (80.5%)	1.94 (1.25–3.02)	0.003*
	Recessive	C/C-C/T	165 (77.5%)	171 (77.7%)	1	
		T/T	48 (22.5%)	49 (22.3%)	0.97 (0.61–1.52)	0.885
	Log-additive	---	---	---	1.28 (0.98–1.69)	0.075
rs3093144	Allele	C	350 (82.5%)	375 (85.2%)	1	
		T	74 (17.5%)	65 (14.8%)	0.82 (0.57–1.18)	0.284
	Codominant	C/C	146 (68.9%)	163 (74.1%)	1	
		C/T	58 (27.4%)	49 (22.3%)	0.76 (0.49–1.19)	0.232
		T/T	8 (3.8%)	8 (3.6%)	0.9 (0.33–2.47)	0.844
	Dominant	C/C	146 (68.9%)	163 (74.1%)	1	
		C/T-T/T	66 (31.1%)	57 (25.9%)	0.78 (0.51–1.19)	0.247
	Recessive	C/C-C/T	204 (96.2%)	212 (96.4%)	1	
		T/T	8 (3.8%)	8 (3.6%)	0.97 (0.36–2.63)	0.951
	Log-additive	---	---	---	0.84 (0.59–1.19)	0.324
rs3093110	Allele	A	369 (86.6%)	402 (91.4%)	1	
		G	57 (13.4%)	38 (8.6%)	0.61 (0.40–0.94)	0.026*
	Codominant	A/A	162 (76.1%)	184 (83.6%)	1	
		A/G	45 (21.1%)	34 (15.5%)	0.67 (0.41–1.11)	IT0.119
		G/G	6 (2.8%)	2 (0.9%)	0.28 (0.06–1.41)	0.123
	Dominant	A/A	162 (76.1%)	184 (83.6%)	1	
		A/G-G/G	51 (23.9%)	36 (16.4%)	0.63 (0.39–1.01)	0.055
	Recessive	A/A-A/G	207 (97.2%)	218 (99.1%)	1	
		G/G	6 (2.8%)	2 (0.9%)	0.3 (0.06–1.51)	0.145
	Log-additive	---	---	---	0.63 (0.41–0.96)	0.034*

HAPE: High altitude pulmonary edema; SNP: Single nucleotide polymorphism; OR: Odds ratio; 95% CI: 95% confidence interval.

*P*-value was calculated by logistic regression analysis with adjustments for age; *P-value < 0.05 indicates statistical significance.

### 3.5. MDR analysis for SNP-SNP interaction in *CYP4F2* with HAPE risk

**Fig 1:**
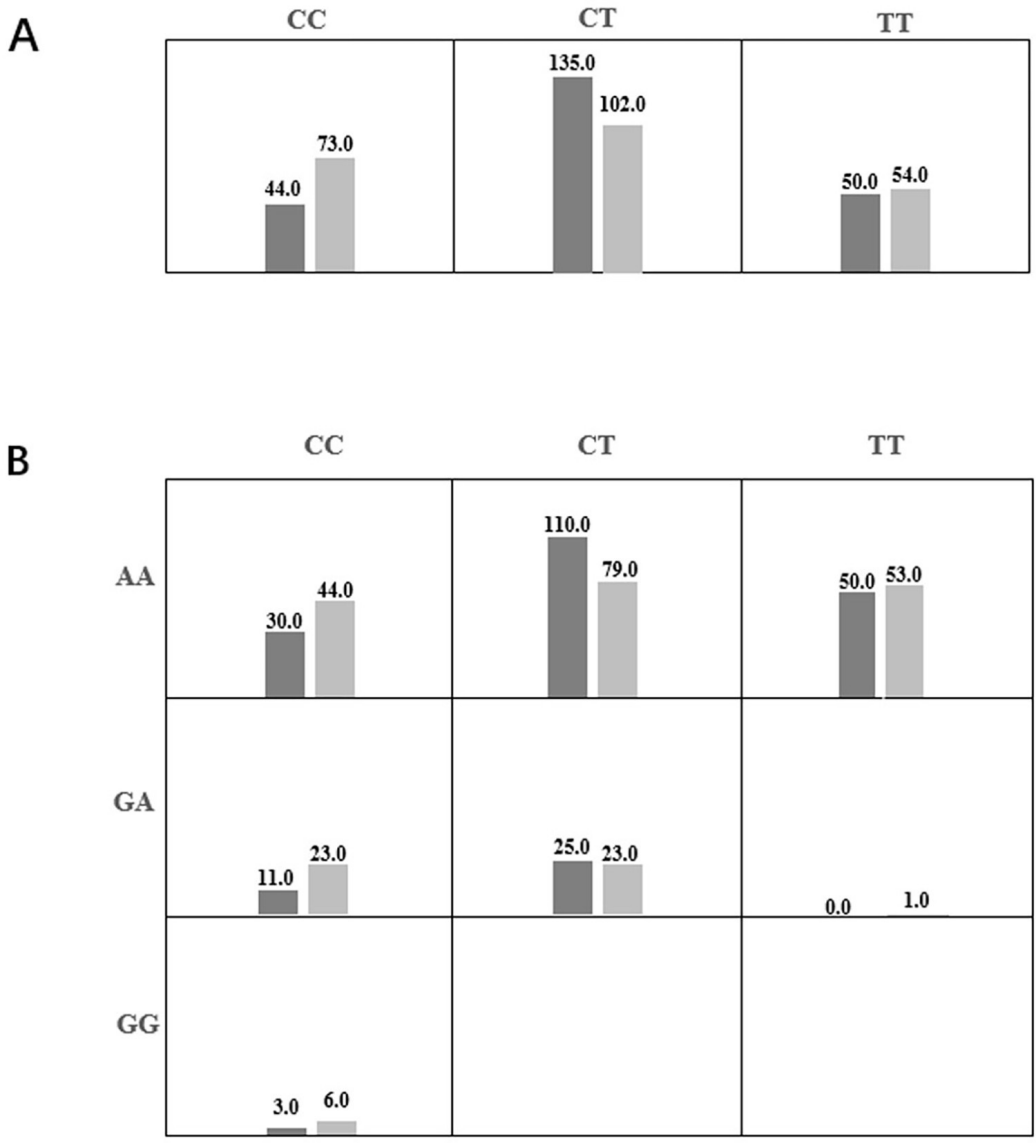
Summary of MDR gene-gene interaction. **(A) S**ingle-locus model. **(B)** Two-locus model. Each cell shows counts of “case” on left and “control” on right.

MDR analysis was used for SNP-SNP interaction in *CYP4F2* with HAPE risk (**Table 8** and **Fig 1**). Rs12459936 was the best single-locus model for HAPE risk (testing accuracy, 0.559; *P* = 0.002; cross-validation consistency, 10/10). Rs12459936rs3093110 was the best multi-loci model with the highest testing accuracy (testing accuracy, 0.5131). **Fig 1** revealed the additive effect between rs12459936-CT and rs3093110-AA on conferring risk towards the susceptibility to HAPE.

**Table 8:** MDR analysis for SNP–SNP interaction in *CYP4F2* with HAPE risk.

Model	Training Bal. Acc.	Testing Bal. Acc.	CVC	OR (95% CI)	*P*
rs12459936	0.5725	0.559	10/10	1.79 (1.23–2.59)	0.002*
rs12459936, rs3093110	0.5754	0.5131	6/10	1.79 (1.23–2.59)	0.002*
rs12459936, rs3093144, rs3093193	0.5786	0.5000	6/10	1.84 (1.27–2.68)	0.0013*
rs12459936, rs3093110, rs3093144, rs3093193	0.5818	0.5000	10/10	1.89 (1.30–2.73)	0.0008*

MDR: Multi-factor dimension reduction; SNP: Single nucleotide polymorphism; Bal. Acc.: Balanced accuracy; CVC: Cross–validation consistency; OR: Odds ratio; CI: Confidence interval. *P* values were calculated using χ^2^ tests. *P-value < 0.05 indicates statistical significance.

### 3.6. FPRP analysis for the association of *CYP4F2* with HAPE risk

FPRP analysis was used to assess whether the important findings were worthy of attention (**Table 9**). At a prior probability level of 0.1, the significant relationship for rs3093193 (FPRP = 0.112, 0.197 and 0.139) and rs12459936 (FPRP = 0.028, and 0.052) was still noteworthy in the overall analysis. The significant finding for rs12459936 (FPRP = 0.181, 0.088, 0.074, and 0.193) in the subgroup at age 32 years remained noteworthy. Moreover, the relationship on rs3093193 (FPRP = 0.088, 0.132 and 0.088) and rs12459936 (FPRP = 0.032 and 0.051) in males was also positive at the prior probability level of 0.1.

**Table 9:** False-positive report probability values for the association of CYP4F2 with HAPE risk.

SNP_ID	Model	OR (95% CI)	*P*	Statistical power	prior probability			
					0.250	0.100	0.010	0.001
**Overall analysis**								
rs3093193	Allele	0.70 (0.52–0.93)	0.014	0.990	0.040*	0.112*	0.581	0.933
	Dominant	0.66 (0.46–0.95)	0.027	0.932	0.075*	0.197*	0.729	0.964
	Log-additive	0.70 (0.52–0.94)	0.017	0.987	0.051*	0.139*	0.640	0.947
rs12459936	Codominant	2.08 (1.33–3.26)	0.001	0.432	0.010*	0.028*	0.243	0.764
	Dominant	1.88 (1.23–2.88)	0.003	0.612	0.018*	0.052*	0.376	0.859
**Subgroup at age ≤ 32 years**								
rs3093193	Allele	0.63 (0.42–0.95)	0.028	0.865	0.087*	0.222	0.759	0.969
	Codominant	0.28 (0.10–0.85)	0.024	0.153	0.326	0.592	0.941	0.994
	Recessive	0.30 (0.10–0.87)	0.027	0.174	0.316	0.580	0.938	0.994
rs12459936	Allele	1.52 (1.06–2.18)	0.022	0.932	0.069*	0.181*	0.708	0.961
	Codominant	2.92 (1.52–5.63)	0.001	0.129	0.031*	0.088*	0.514	0.914
	Codominant	2.30 (1.10–4.83)	0.027	0.356	0.190*	0.413	0.885	0.987
	Dominant	2.69 (1.45–4.96)	0.002	0.171	0.026*	0.074*	0.469	0.899
	Log-additive	1.52 (1.05–2.19)	0.026	0.930	0.074*	0.193*	0.724	0.964
**Subgroup at age >32 years**								
rs3093193	Codominant	0.57 (0.33–0.99)	0.046	0.679	0.169*	0.379	0.870	0.985
rs3093110	Allele	0.53 (0.29–0.96)	0.034	0.576	0.159*	0.361	0.861	0.984
	Codominant	0.42 (0.21–0.83)	0.013	0.308	0.109*	0.268	0.801	0.976
	Dominant	0.44 (0.23–0.86)	0.016	0.354	0.122*	0.293	0.820	0.979
	Log-additive	0.52 (0.29–0.95)	0.034	0.551	0.154*	0.353	0.857	0.984
**Males**								
rs3093193	Allele	0.67 (0.50–0.91)	0.010	0.970	0.031*	0.088*	0.514	0.914
	Codominant	0.65 (0.44–0.97)	0.036	0.901	0.104*	0.259	0.793	0.975
	Dominant	0.62 (0.42–0.91)	0.015	0.864	0.048*	0.132*	0.626	0.944
	Log-additive	0.67 (0.50–0.91)	0.011	0.970	0.031*	0.088*	0.514	0.914
rs12459936	Codominant	2.12 (1.33–3.37)	0.002	0.403	0.011*	0.032*	0.267	0.787
	Dominant	1.94 (1.25–3.02)	0.003	0.554	0.018*	0.051*	0.374	0.858
rs3093110	Allele	0.61 (0.40–0.94)	0.026	0.816	0.084*	0.216	0.752	0.968
	Log-additive	0.63 (0.41–0.96)	0.034	0.859	0.099*	0.249	0.784	0.973

Statistical power was calculated using the number of observations in the subgroup and the OR and *P* values in this table.

*The level of false-positive report probability threshold was set at 0.2, and noteworthy findings were presented.

### 3.7. Gene interaction, gene ontology, and KEGG analysis

**Fig 2:**
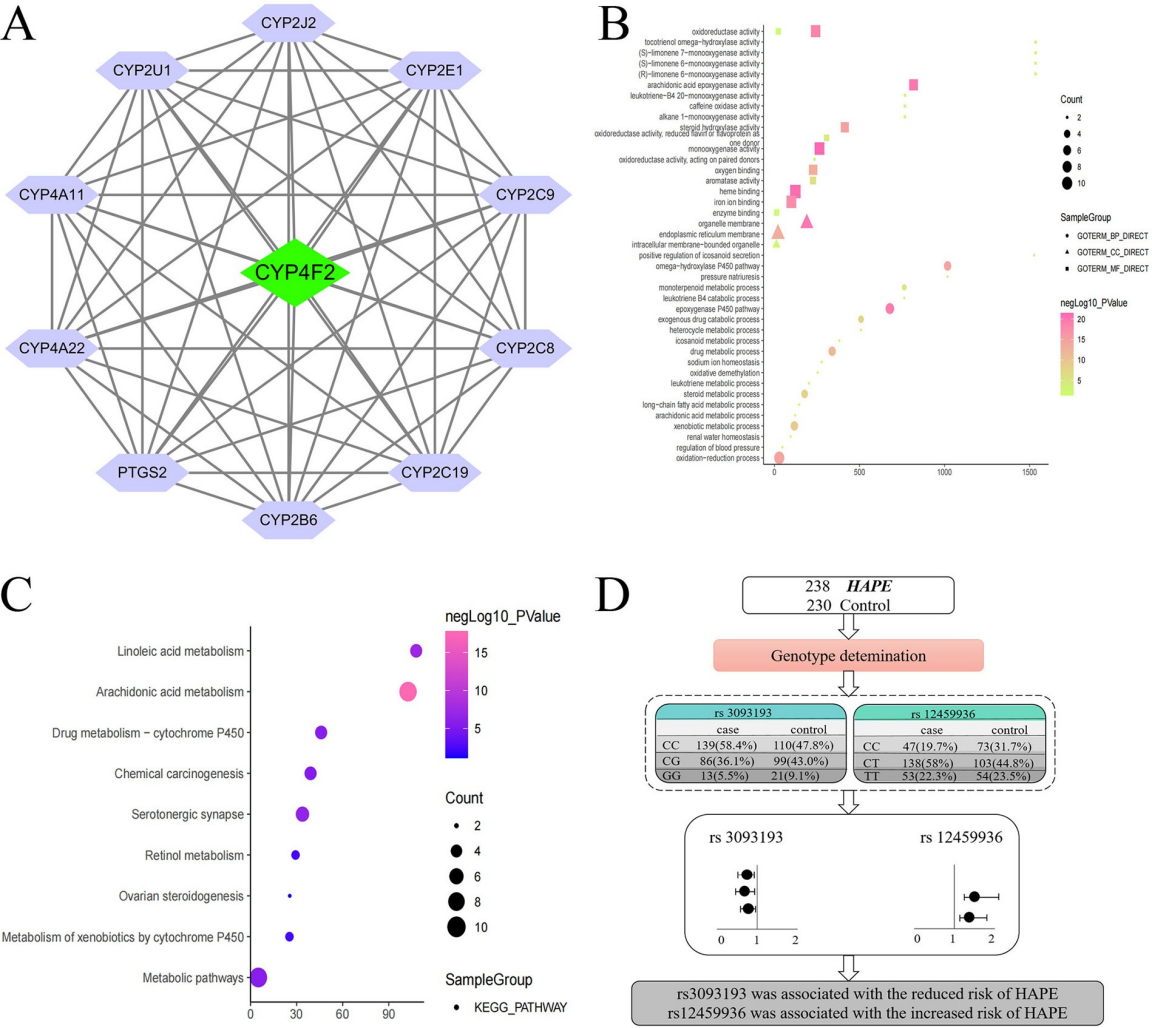
Functional enrichment analysis for *CYP4F2* related genes. **(A)** The interaction of *CYP4F2* with other protein (top 10). The yellow circle represents the *CYP4F2*, while red circles delineate other proteins. **(B)** The KEGG analysis. The KEGG pathway with corresponding adjusted p-values analyzed by clusterProfiler, which are displayed in a dot plot. The color scales indicated the different thresholds of adjusted p-values, and the sizes of the dots represented the gene count of each term. **(C)** Gene ontology analysis. The x-axis shows significantly enriched ‘biological process (green)’ categories, ‘cellular component (orange)’ categories and ‘molecular function (purple)’ categories associated with the targets; the y-axis shows the enrichment scores (*P* value) of these terms. (**D**) The flow chart. The study recruited 238 patients with HAPE and 230 healthy controls. Rs3093193 was shown to reduce the risk of HAPE, while rs12459936 was increased the susceptibility to HAPE.

**Fig 2** showed the results of functional enrichment analysis. The interaction of *CYP4F2* with other protein (top 10) was displayed in **Fig 2A**. Go analysis results are divided into three parts: biological process, cellular component, and molecular function. The possible function of *CYP4F2* was related to omega-hydroxylase P450 pathway, regulation of blood pressure, organelle membrane, heme binding, iron ion binding, and oxidoreductase activity (**Fig 2B** and **2C**).

**S1 Fig** displayed the results of functional enrichment analysis for HAPE susceptible genes and *CYP4F2* related genes. PPI results (**S1A Fig**) displayed that there was interaction between these genes. These HAPE susceptible genes may participate in the CYP gene related signal pathway and *CYP4F2* may also participate in the related inflammatory through *CYP2E1* and *CYP11B2*. These studies suggest that *CYP2E1* and *CYP11B2* may play an important role in the pathogenesis of HAPE, and we will further study the correlation between polymorphisms in these genes and HAPE susceptibility. KEGG analysis displayed that these genes were associated with arachidonic acid metabolism, linoleic acid metabolism, AGE-RAGE signaling pathway in diabetic complications, HIF-1 signaling pathway, fluid shear stress and atherosclerosis, retinol metabolism, drug metabolism—cytochrome P450, vascular smooth muscle contraction, hypertrophic cardiomyopathy (HCM), and pathways in cancer (**S1B and S1C Fig**).

## 4. Discussion

In the study, rs3093193 was shown to reduce the risk of HAPE, while rs12459936 conferred an increased susceptibility to HAPE in the Chinese Han population (**Fig 2D**). Age stratified analysis revealed that rs3093193 and rs12459936 were correlated with the HAPE risk in the subgroup at age ≤ 32 years, and rs3093193 and rs3093110 were correlated with the HAPE risk at age > 32 years. In males, rs3093193, rs12459936, and rs3093110 were related to HAPE risk. SNP-SNP interaction analysis revealed the additive effect between rs12459936-CT and rs3093110-AA on conferring risk towards the susceptibility to HAPE. A comprehensive search in PubMed, Embase, and the China National Knowledge Infrastructure databases was conducted using the search terms: “*CYP4F2* and HAPE/ High altitude pulmonary edema”. As far as we know, this is the first study to evaluate the correlation of *CYP4F2* SNPs with HAPE risk in the Chinese Han population.

Severe hypoxia induced by HAPE is prone to immunogenic changes and involvement in the development of HAPE, leading to significant changes in the prevalence of immune function during HAPE [19]. Inflammatory markers of leukotriene B4, arachidonic acid, CRP, and CCR5 are upregulated in response to high altitude, and hypoxia-induced inflammation at high altitude may contribute to the development of HAPE [20,21]. The *CYP4F2* gene polymorphisms, known as a major member of the CYP450 subfamily, is located on chromosome 19p13.12 and encodes o-hydroxylase. CYP4F2 is the major catalyst of 20-HETE formation in human liver and kidney microsomes. In addition to CYP4Fs catalyzing the formation of 20-HETE, these enzymes also ω-hydroxylate and deactivate proinflammatory 5-, 8-, and 12-HETE, suggesting the dual roles for the P450 ω-hydroxylases in both the initiation and resolution phases of inflammation [22]. 20-HETE protects pulmonary vascular endothelial cells (PMVEC) under normoxia and hypoxia [23]. CYP4Fs/20-HETE has been reported to enhance angiogenesis, pulmonary vascular tone, and endothelial nitric oxide synthase function [24]. These suggested that *CYP4F2* might have an important pathogenesis on HAPE.

HAPE is a potentially fatal disease caused by nonuniform hypoxic pulmonary vasoconstriction, leading to pulmonary capillary stress failure and a high-permeability pulmonary edema [25]. A previous study showed that some individuals are more susceptible to HAPE than others when exposed to identical hypoxic conditions, suggesting that genetic susceptibility might contribute to an individual’s risk of HAPE [26]. Several genetic studies have demonstrated that a genetic susceptibility may play a vital role in the development of HAPE, such as *ACE*, *EDN1*, *ACYP2*, *RTEL1*, and *VEGF* [27,28]. It is reported that some SNPs in *CYP4F2* gene, such as rs3093105, rs3093135, rs3093200, rs1558139, and rs2108622, can cause an increase or decrease risk in o-hydroxylase activity, which results in altered levels of 20-HETE production [29]. Studies have also shown that rs1558139 and rs2108622 can lead to an increased or decreased activity of the *CYP4F2* gene [30]. Furthermore, studies show that SNPs of the *CYP4F2* gene are associated with various diseases, such as hypertension [31,32], cerebral infarction [33], myocardial infarction [34–36], and metabolic syndrome [37]. In the present case-control study, our results first showed that rs3093193 and rs12459936 in *CYP4F2* were linked to the risk of HAPE in the Chinese Han population. Based on HaploReg database, rs3093193 and rs12459936 might be related to the regulation of enhancer histone, motifs changed, and/or selected eQTL hits. Considering the established function of SNPs and their influence on gene expression, we speculated that SNPs may affect the occurrence risk of HAPE by changing the expression of *CYP4F2* or its o-hydroxylase activity. However, the mechanisms still need more functional studies to testify.

Age is not a risk factor for altitude illness, and in fact, it may be protective against severe altitude illness [38]. Age stratified analysis revealed that rs3093193 was correlated with a decreased HAPE risk in both the subgroup with age ≤ 32 years and > 32 years. Moreover, rs12459936 was related to enhance the HAPE risk in people at age ≤ 32 years, while rs3093110 was associated with a decreased HAPE risk in people at age > 32 years. Male may be at greater risk for HAPE [39]. In males, rs3093193 and rs3093110 were correlated with a decreased risk of HAPE, while rs12459936 improved. These findings may suggest that genetic susceptibility to HAPE differs by age and sex, and emphasizes the importance of considering heterogeneity in hereditary and HAPE association studies.

A previous study reported a genome-wide association study of high-altitude pulmonary edema in Han Chinese, and GO and pathway enrichment analysis displayed that these genes were significantly correlated with arachidonic acid metabolism and other metabolism [40]. A study found that plasma retinol binding precursor showed overexpression in HAPE patients as compared to controls [41], suggesting retinol metabolism related inflammatory response system might be linked to the pathophysiology of HAPE. Through gene interaction and functional enrichment analysis, we found that *CYP4F2* gene was related to arachidonic acid metabolism and retinol metabolism, suggesting that *CYP4F2* might be involved in the pathogenesis of HAPE. Although we first investigated the association of rs3093193, rs12459936 and rs3093110 in *CYP4F2* on HAPE susceptibility, there are still some potential limitations. First and foremost, all participants were recruited from the identical hospitals, which may result in a selection offset. Next, the number of cases in our study was not huge and sufficient, and our study population was all Chinese Han people which can’t preclude false-negative results and cannot be extrapolated to other populations. Then, *CYP4F2* gene polymorphisms may be related to the development of HAPE, but the mechanisms had not been studied. Further functional studies and a large number of well-designed studies are still needed to further clarify the effect of *CYP4F2* polymorphisms on HAPE. Finally, due to the lack of information on the comorbidities, this study failed to assess the association of genetic variants with HAPE comorbidities. In the future, we would like to enlarge sample size and complete these data to evaluate the relationship.

In conclusion, our study is the first to offer some useful and substantial information on the association between *CYP4F2* gene polymorphisms and HAPE risk in the Chinese Han population, which may provide new data to facilitate earlier diagnosis and promote early prevention and control, and shed light on the new candidate genes and new ideas for the study.

## Supporting information

S1 Fig Functional enrichment analysis for HAPE susceptible genes and *CYP4F2* related genes. **(A)** The protein and protein interaction of HAPE susceptible genes and *CYP4F2* related genes. **(B)** Gene ontology analysis. The x-axis shows significantly enriched GO categories associated with the targets; the y-axis shows the enrichment scores (*P* value) of these terms. **(C)** The KEGG analysis. The KEGG pathway with corresponding adjusted p-values analyzed by clusterProfiler. The color scales indicated the different thresholds of adjusted p-values.
(TIF)

## References

[1] Mishra A, Ali Z, Vibhuti A, Kumar R, Alam P, Ram R, et al. CYBA and GSTP1 variants associate with oxidative stress under hypobaric hypoxia as observed in high-altitude pulmonary oedema. Clinical science (London, England: 1979). 2012;122(6):299–309. doi: 10.1042/CS20110205 21973220

[2] Askew EW. Work at high altitude and oxidative stress: antioxidant nutrients. Toxicology. 2002;180(2):107–19. doi: 10.1016/s0300-483x(02)00385-2 12324188

[3] Pennardt A. High-altitude pulmonary edema: diagnosis, prevention, and treatment. Current sports medicine reports. 2013;12(2):115–9. doi: 10.1249/JSR.0b013e318287713b 23478563

[4] Scherrer U, Rexhaj E, Jayet PY, Allemann Y, Sartori C. New insights in the pathogenesis of high-altitude pulmonary edema. Progress in cardiovascular diseases. 2010;52(6):485–92. doi: 10.1016/j.pcad.2010.02.004 20417341

[5] Rong H, He X, Zhu L, Zhu X, Kang L, Wang L, et al. Association between regulator of telomere elongation helicase1 (RTEL1) gene and HAPE risk: A case-control study. Medicine. 2017;96(39):e8222. doi: 10.1097/MD.0000000000008222 28953687 PMC5626330

[6] Zhu L, Liu L, He X, Yan M, Du J, Yang H, et al. Association between genetic polymorphism of telomere-associated gene ACYP2 and the risk of HAPE among the Chinese Han population: A Case-control study. Medicine. 2017;96(13):e6504. doi: 10.1097/MD.0000000000006504 28353602 PMC5380286

[7] Srivastava S, Pandey H, Singh SK, Tripathi YB. Anti-oxidant, anti-apoptotic, anti-hypoxic and anti-inflammatory conditions induced by PTY-2 against STZ-induced stress in islets. Bioscience trends. 2019;13(5):382–93. doi: 10.5582/bst.2019.01181 31597821

[8] He X, Wang L, Zhu L, Yuan D, He Y, Jin T. A case-control study of the genetic polymorphism of IL6 and HAPE risk in a Chinese Han population. 2018;12(9):2419–25. 10.1111/crj.12922 30074683

[9] Chen X, Zhang Q, Wang J, Xin Z, Chen J, Luo W. Combined machine learning and functional magnetic resonance imaging allows individualized prediction of high-altitude induced psychomotor impairment: The role of neural functionality in putamen and pallidum. Bioscience trends. 2019;13(1):98–104. doi: 10.5582/bst.2019.01002 30814403

[10] Hilty MP, Zügel S, Schoeb M, Auinger K, Dehnert C, Maggiorini M. Soluble Urokinase-Type Plasminogen Activator Receptor Plasma Concentration May Predict Susceptibility to High Altitude Pulmonary Edema. Mediators of inflammation. 2016;2016:1942460. doi: 10.1155/2016/1942460 27378823 PMC4917741

[11] Gilroy DW, Edin ML. CYP450-derived oxylipins mediate inflammatory resolution. 2016;113(23):E3240–9. doi: 10.1073/pnas.1521453113 27226306 PMC4988604

[12] Alvarellos ML, Sangkuhl K, Daneshjou R, Whirl-Carrillo M, Altman RB, Klein TE. PharmGKB summary: very important pharmacogene information for CYP4F2. Pharmacogenetics and genomics. 2015;25(1):41–7. doi: 10.1097/FPC.0000000000000100 25370453 PMC4261059

[13] Aires V, Hichami A, Boulay G, Khan NA. Activation of TRPC6 calcium channels by diacylglycerol (DAG)-containing arachidonic acid: a comparative study with DAG-containing docosahexaenoic acid. Biochimie. 2007;89(8):926–37. doi: 10.1016/j.biochi.2006.10.016 17532549

[14] Cheng J, Edin ML, Hoopes SL, Li H, Bradbury JA, Graves JP, et al. Vascular characterization of mice with endothelial expression of cytochrome P450 4F2. FASEB journal: official publication of the Federation of American Societies for Experimental Biology. 2014;28(7):2915–31. doi: 10.1096/fj.13-241927 24668751 PMC4062824

[15] Ding Y, Yang Y, Li Q, Feng Q, Xu D, Wu C, et al. The correlation between CYP4F2 variants and chronic obstructive pulmonary disease risk in Hainan Han population. Respiratory research. 2020;21(1):86. doi: 10.1186/s12931-020-01348-6 32295578 PMC7161254

[16] He R, Li M, Li A, Dang W, Yang T, Li J, et al. CYP4F2 and CYP3A5 gene polymorphisms and lung cancer in Chinese Han population. 2020;20(3):461–8. 10.1007/s10238-020-00631-6 PMC7366610 32350633

[17] Zhuo ZJ, Liu W, Zhang J, Zhu J, Zhang R, Tang J, et al. Functional Polymorphisms at ERCC1/XPF Genes Confer Neuroblastoma Risk in Chinese Children. EBioMedicine. 2018;30:113–9. doi: 10.1016/j.ebiom.2018.03.003 29544698 PMC5952228

[18] Jin T, Yang H, Zhang J, Yunus Z, Sun Q, Geng T, et al. Polymorphisms and phenotypic analysis of cytochrome P450 3A4 in the Uygur population in northwest China. International journal of clinical and experimental pathology. 2015;8(6):7083–91. 26261601 PMC4525935

[19] Shukla D, Saxena S, Purushothaman J, Shrivastava K, Singh M, Shukla S, et al. Hypoxic preconditioning with cobalt ameliorates hypobaric hypoxia induced pulmonary edema in rat. European journal of pharmacology. 2011;656(1–3):101–9. doi: 10.1016/j.ejphar.2011.01.038 21296072

[20] Bao Y, Yang J, Duan Y, Chen Y, Chen W, Sun D. The C-reactive protein to albumin ratio is an excellent prognostic predictor for gallbladder cancer. Bioscience trends. 2021;14(6):428–35. doi: 10.5582/bst.2020.03326 33239498

[21] Rosenberger P, Schwab JM, Mirakaj V, Masekowsky E, Mager A, Morote-Garcia JC, et al. Corrigendum: Hypoxia-inducible factor-dependent induction of netrin-1 dampens inflammation caused by hypoxia. Nature immunology. 2015;16(5):544. doi: 10.1038/ni0515-544a 25898201

[22] Johnson AL, Edson KZ, Totah RA, Rettie AE. Cytochrome P450 ω-Hydroxylases in Inflammation and Cancer. Advances in pharmacology (San Diego, Calif). 2015;74:223–62. 26233909 10.1016/bs.apha.2015.05.002 PMC4667791

[23] Sugumaran P, Narayanan V, Zhu D, Medhora M, Jacobs ER, Chandramohan Y, et al. Prophylactic supplementation of 20-HETE ameliorates hypoxia/reoxygenation injury in pulmonary vascular endothelial cells by inhibiting apoptosis. Acta histochemica. 2020;122(1):151461. doi: 10.1016/j.acthis.2019.151461 31706620

[24] Medhora M, Chen Y, Gruenloh S, Harland D, Bodiga S, Zielonka J, et al. 20-HETE increases superoxide production and activates NAPDH oxidase in pulmonary artery endothelial cells. American journal of physiology Lung cellular and molecular physiology. 2008;294(5):L902–11. doi: 10.1152/ajplung.00278.2007 18296498 PMC2586843

[25] Hartman-Ksycińska A, Kluz-Zawadzka J, Lewandowski B. High altitude illness. Przeglad epidemiologiczny. 2016;70(3):490–9. 27888818

[26] Qi Y, Niu WQ, Zhu TC, Liu JL, Dong WY, Xu Y, et al. Genetic interaction of Hsp70 family genes polymorphisms with high-altitude pulmonary edema among Chinese railway constructors at altitudes exceeding 4000 meters. Clinica chimica acta; international journal of clinical chemistry. 2009;405(1–2):17–22. doi: 10.1016/j.cca.2009.03.056 19351530

[27] He Y, Zhang X, Li X, Du J, He X, Zhang Z, et al. Telomere length-related gene ACYP2 polymorphism is associated with the risk of HAPE in Chinese Han population. The journal of gene medicine. 2016;18(9):244–9. doi: 10.1002/jgm.2896 27552709

[28] He Y, Liu L, Xu P, He N, Yuan D, Kang L, et al. Association between single nucleotide polymorphisms in ADRB2, GNB3 and GSTP1 genes and high-altitude pulmonary edema (HAPE) in the Chinese Han population. Oncotarget. 2017;8(11):18206–12. doi: 10.18632/oncotarget.15309 28212552 PMC5392320

[29] Stec DE, Roman RJ, Flasch A, Rieder MJ. Functional polymorphism in human CYP4F2 decreases 20-HETE production. Physiological genomics. 2007;30(1):74–81. doi: 10.1152/physiolgenomics.00003.2007 17341693

[30] Luo XH, Li GR, Li HY. Association of the CYP4F2 rs2108622 genetic polymorphism with hypertension: a meta-analysis. Genetics and molecular research: GMR. 2015;14(4):15133–9. doi: 10.4238/2015.November.25.1 26634476

[31] Ward NC, Tsai IJ, Barden A, van Bockxmeer FM, Puddey IB, Hodgson JM, et al. A single nucleotide polymorphism in the CYP4F2 but not CYP4A11 gene is associated with increased 20-HETE excretion and blood pressure. Hypertension (Dallas, Tex: 1979). 2008;51(5):1393–8. doi: 10.1161/HYPERTENSIONAHA.107.104463 18391101

[32] Fan F, Ge Y, Lv W, Elliott MR, Muroya Y, Hirata T, et al. Molecular mechanisms and cell signaling of 20-hydroxyeicosatetraenoic acid in vascular pathophysiology. Frontiers in bioscience (Landmark edition). 2016;21:1427–63. doi: 10.2741/4465 27100515 PMC5064940

[33] Fu Z, Nakayama T, Sato N, Izumi Y, Kasamaki Y, Shindo A, et al. A haplotype of the CYP4F2 gene is associated with cerebral infarction in Japanese men. American journal of hypertension. 2008;21(11):1216–23. doi: 10.1038/ajh.2008.276 18787519

[34] Yan HQ, Yuan Y, Zhang P, Huang Z, Chang L, Gui YK. CYP4F2 gene single nucleotide polymorphism is associated with ischemic stroke. Genetics and molecular research: GMR. 2015;14(1):659–64. doi: 10.4238/2015.January.30.8 25730002

[35] Fava C, Montagnana M, Almgren P, Rosberg L, Lippi G, Hedblad B, et al. The V433M variant of the CYP4F2 is associated with ischemic stroke in male Swedes beyond its effect on blood pressure. Hypertension (Dallas, Tex: 1979). 2008;52(2):373–80. doi: 10.1161/HYPERTENSIONAHA.108.114199 18574070

[36] Deng S, Zhu G, Liu F, Zhang H, Qin X, Li L, et al. CYP4F2 gene V433M polymorphism is associated with ischemic stroke in the male Northern Chinese Han population. Progress in neuro-psychopharmacology & biological psychiatry. 2010;34(4):664–8. doi: 10.1016/j.pnpbp.2010.03.009 20227456

[37] Tabur S, Oztuzcu S, Oguz E, Demiryürek S, Dagli H, Alasehirli B, et al. CYP gene expressions in obesity-associated metabolic syndrome. Obesity research & clinical practice. 2016;10(6):719–23. doi: 10.1016/j.orcp.2016.03.001 27010496

[38] Richalet JP, Lhuissier FJ. Aging, Tolerance to High Altitude, and Cardiorespiratory Response to Hypoxia. High altitude medicine & biology. 2015;16(2):117–24. doi: 10.1089/ham.2015.0030 25946570

[39] Jensen JD, Vincent AL. High Altitude Pulmonary Edema. StatPearls. Treasure Island (FL): StatPearls Publishing Copyright © 2021,. StatPearls Publishing LLC. 2021.

[40] Yang YZ, Wang YP, Ma L, Du Y, Ge RL. [Genome-wide association study of high-altitude pulmonary edema in Han Chinese]. Yi chuan = Hereditas. 2013;35(11):1291–9. doi: 10.3724/sp.j.1005.2013.01291 24579312

[41] Ahmad Y, Shukla D, Garg I, Sharma NK, Saxena S, Malhotra VK, et al. Identification of haptoglobin and apolipoprotein A-I as biomarkers for high altitude pulmonary edema. Functional & integrative genomics. 2011;11(3):407–17. doi: 10.1007/s10142-011-0234-3 21755356

