# Supplementary figures and images for: Suggestive evidence of CYP4F2 gene polymorphisms with HAPE susceptibility in the Chinese Han population
Source: PLoS One. 2023 Jan 12;18(1):e0280136. doi: 10.1371/journal.pone.0280136 (PMC9836295; doi:10.1371/journal.pone.0280136)

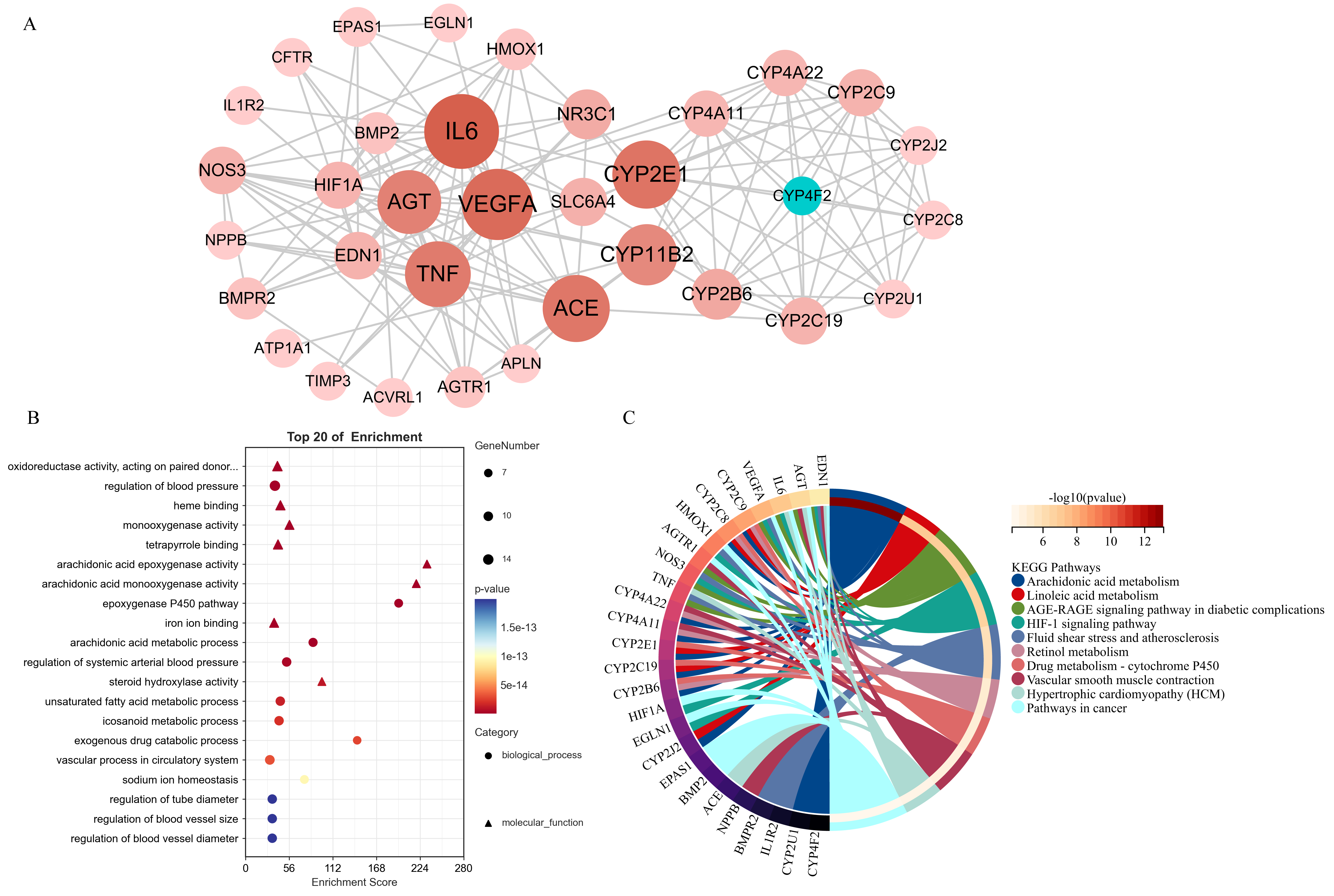

Supplement: S1 Fig — (A) The protein and protein interaction of HAPE susceptible genes and CYP4F2 related genes. (B) Gene ontology analysis. The x-axis shows significantly enriched GO categories associated with the targets; the y-axis shows the enrichment scores (P value) of these terms. (C) The KEGG analysis. The KEGG pathway with corresponding adjusted p-values analyzed by clusterProfiler. The color scales indicated the different thresholds of adjusted p-values. (TIF) [file pone.0280136.s001.tif]
